# Positive rumination can (also) interfere with sleep: A study in a non-clinical sample

**DOI:** 10.3389/fpsyt.2022.889810

**Published:** 2022-08-09

**Authors:** Ilana S. Hairston, Lilach Portal, Tal Carmon

**Affiliations:** ^1^Psychology Department, Tel Hai Academic College, Kiryat Shmona, Israel; ^2^The Institute of Information Processing and Decision Making (IIPDM), University of Haifa, Haifa, Israel; ^3^Psychology Department, The Max Stern Yezreel Valley College, Kiryat Shmona, Israel

**Keywords:** suppression (psychology), reappraisal, insomnia, affect regulation, cognitive arousal

## Abstract

It is postulated that negative ruminations perpetuate insomnia symptoms by increasing arousal. Less is known about the role of positive rumination. In this study, we set out to test the association between positive and negative ruminations and insomnia symptoms in a non-clinical sample, asking whether reappraisal and suppression moderate the relationship between rumination types and symptoms of insomnia.

**Methods:**

A convenience sample of 354 participants (59% women), ages 18–50, responded to online questionnaires regarding symptoms of insomnia (Insomnia Severity Index [ISI]), Emotion Regulation Questionnaire that provides separate scales for Reappraisal and Suppression, Negative Rumination (Ruminative Response Scale), Positive Rumination and Dampening (Responses to Positive Affect questionnaire), and general health and demographics.

**Results:**

About 30% of respondents had moderate to severe symptoms of insomnia according to the ISI. The primary hypothesis was tested using three moderation models, where rumination type, emotion regulation styles, and interaction terms were predictors, and ISI scores were the outcome variable. Negative rumination positively predicted ISI (β = 0.56, *p* < 0.001), while the interaction terms with Reappraisal (β = 0.02, *p* = 0.575) and Suppression (β = 0.07, *p* = 0.092) were not significant. Dampening also positively predicted ISI (β = 0.56, *p* < 0.001), with the interaction term with Reappraisal nearly significant (β = −0.09, *p* = 0.060), but not with Suppression (β = 0.08, *p* =0.098). Positive rumination negatively predicted ISI (β = −0.12, *p* = 0.021), this relationship was reversed with emotion regulation factors in the model (β = 0.11, *p* = 0.094), where the interaction with Reappraisal (β = 0.13, *p* = 0.020) and Suppression (β = −0.13, *p* = 0.024) were both significant.

**Discussion:**

Positive Rumination weakly and negatively correlated with ISI, but the combination with Reappraisal was associated with more insomnia symptoms. By contrast, Dampening was associated with more insomnia symptoms, with minimal to no moderating effects. These observations are interpreted in the context of the role of emotion regulation strategies and sleep, and their potential clinical implications.

## Introduction

Symptoms of insomnia are highly prevalent with about 30–50% of adults reporting sleep difficulty per year (1, 2), with long-term implications for mental (3) and physical health (4). According to the American Academy of Sleep Medicine, insomnia is defined as the persistent difficulty with sleep initiation, duration, consolidation, or quality, despite adequate conditions for sleep, resulting in some form of daytime impairment (5), and is rarely associated with objective measures (6). Daytime impairments may include fatigue, hyperarousal, and negative affective states (7, 8). Psychophysiological factors, such as stress, worry and hyperarousal, play a major role in poor sleep (9). Specifically, the harmful impact of negative over-thinking such as worry and rumination is well established in both insomnia patients and non-clinical samples (10). For example, in a large population survey “worry/thinking” was the most common reason for sleep problems [37.9%, (11)]. Similarly, in a large study of daytime employees, Kompier et al. (12) found that high levels of work-related rumination constituted the strongest predictor of sleep complaints.

Negative rumination is a core transdiagnostic cognitive process implicated in several clinical disorders (13) including insomnia (10). It is defined as a maladaptive form of emotion regulation comprising repetitive self-reflection focused on problematic situations or events, their evoked emotions and their consequences (14). Negative ruminations prolong negative affective states by focusing attention on negative cognitions and arrogating attentional resources (15). For example, the association between self-reported stress and severity of insomnia symptoms was amplified in individuals with a tendency to ruminate at bedtime (16). Accordingly, negative ruminations increase arousal, which - in turn - interferes with subsequent sleep and sleep quality (17–19). Further, Koster et al. (20) suggested that the underlying mechanism of rumination is failure to disengage working memory from negative material [see also Zetsche et al. (21)].

Though the plurality of investigations on rumination center on sad or depressed mood, recent focus has shifted to rumination with positive valence. Positive rumination is defined as self-referential thoughts regarding positive events and experiences. Feldman et al. (22) distinguished between two types of positive ruminations: Thoughts focused on positive experiences (of self or the emotion) that maintain and/or enhance positive affective states (Positive Rumination), and cognitions aimed at dampening positive mood (Dampening). Positive Ruminations boost positive mood (23, 24) and decrease depressive symptoms and negative mood (22, 25, 26), whereas Dampening has been associated with dysphoria and anhedonia (27–29). Notably, positive ruminations have also been associated with manic symptoms and episode frequency in patients with bipolar spectrum disorders (30–32) which raises the possibility that ruminating about positive experiences may have an arousing effect similar to negative ruminations [e.g., (33)].

Arguably, the current state-of-the-knowledge is unclear as to the executive processes underlying positive rumination. One possibility is that, like negative rumination, positive rumination reflects a tendency toward perseverative and intrusive thinking. This would be consistent with the above-mentioned associations with manic symptoms and would be independent of whether the cognitions are focused on maintaining the positive affective state or dampening it. Support for this view can be found in factor analytic studies, across various measures of rumination, demonstrating an overall tendency for repetitive thoughts, both positive and negative [reviewed in Smith and Alloy (34)]. In this formulation, ruminating about either positive or negative experiences is an involuntary and intrusive cognitive process that increases emotional arousal, even when the intention is to reduce it (i.e., dampening).

The downstream effects of positive rumination on arousal, and therefore on sleep, have not, to our knowledge, been assessed in non-clinical samples. Trait positive affect has been reliably associated with better subjective measures of sleep (35), consistent with reports that the induction of positive emotions increases vagal tone (36, 37), which is associated with better sleep (38–40). Importantly, Talbot et al. (41) found that a positive mood induction manipulation delayed sleep onset among euthymic bipolar participants, and hastened sleep onset among controls. Further, Kreibig (42) demonstrated that different positive emotions elicit different physiological responses, where some decrease while others increase sympathetic arousal. Thus, positive ruminations may have different effects on sleep depending on the emotion involved and the population tested.

As arousal due to rumination interferes with sleep, a person may wish to down-regulate this arousal using other emotion regulation strategies. For this investigation, we focused on the potential moderating effects of two additional emotion regulation strategies: Reappraisal and Suppression. Reappraisal is a cognitive strategy that involves redefining potentially affect-eliciting situations to change the emotional experience. Suppression is a form of response modulation involving the inhibition of ongoing emotion expression (43). Both are viewed as conscious, goal-oriented strategies, although they target different aspects of the emotional experience: Reappraisal is focused on emotion-related knowledge, whereas suppression targets bodily responses (44). On the trait level, habitual reappraisal is considered a healthy emotion regulation strategy and is associated with reduced negative and enhanced positive affect, and better general wellbeing. In contrast, habitual suppression is associated with lower negative and positive affect but with elevated arousal (45, 46).

Experimentally, reappraisal has been shown to effectively down-regulate both positive and negative emotional arousal [e.g., (44, 47)]. The effects of suppression are more complex. Engaging in expressive suppression decreases negative emotional experiences (48, 49); however physiological arousal may increase (50) or fail to decrease (51). Both strategies require executive control: When participants are asked to regulate their emotions using either reappraisal or suppression, activation in executive-related brain regions is observed (50, 52). Importantly, executive control training has been found to enhance the use of reappraisal when processing information that elicits negative emotions, to reduce rumination and/or associated negative emotions (53, 54).

**Figure 1:**
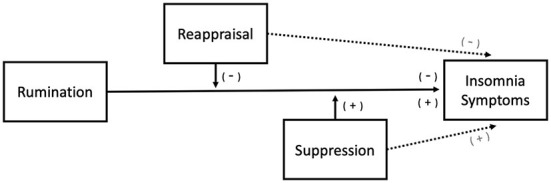
Proposed moderation model. Negative Rumination and Dampening are predicted to increase subjective sleep difficulty whereas two predictions (either increase or decrease) for Positive Rumination are possible. Reappraisal is predicted to attenuate the effects of rumination whereas Suppression is expected to enhance the effects. Additionally, Reappraisal is independently expected to reduce subjective sleep difficulty, and Suppression to increase.

Based on the above, the rationale of the present study was that ruminations, with either positive or negative content, reflect loss of executive control over conscious thoughts, which will increases arousal for negative affective states (i.e., negative rumination and dampening), and may either increase or decrease arousal for positive rumination. Therefore we hypothesize that (1) in line with previous reports negative rumination [e.g., (10)] and dampening (33) will correlate with more insomnia symptoms. As the tendency to engage in reappraisal may reinstate control of ruminative cognitions we hypothesize that (2) reappraisal will attenuate the association between negative rumination and dampening with insomnia symptoms. As suppression, as an emotion regulation strategy tends to exacerbate negative affect and increase arousal we hypothesize (3) that suppression will enhance the association between negative rumination and dampening with insomnia symptoms. Given the possible divergent effects of positive emotions on arousal, we leave the possibility open that (4) positive rumination will either attenuate or increase self-reported sleep disturbances; (5) that reappraisal will attenuate and (6) suppression will enhance the associations of rumination with insomnia symptoms (see Figure 1).

## Methods

### Participants

A convenience sample of 406 respondents answered the web-based survey administered between May and July of 2019, of whom 352 completed all questionnaires. One was removed due to non-valid responses (see statistical analysis). Ages ranged 18–50 (*M* = 30.4 ± 6.0) and 58.7% were women. Eleven reported having one or more physical health diagnosis, which included diabetes (*n* = 3), high blood pressure (*n* = 7) and heart disease (*n* = 2). Sixty endorsed having one or more mental health diagnosis, which included attention deficit disorder (*n* = 43), addictions (*n* = 2), anxiety disorder (*n* = 20), depression disorder (*n* = 7), eating disorders (*n* = 3), and sleep disorder (*n* = 11). Twenty-two reported taking medications, which included antidepressants (*n* = 6), contraceptives (*n* = 3), allergy (*n* = 2), blood pressure (*n* = 3), migraine (*n* = 1), and insulin (*n* = 1). Seven did not disclose. Sample demographics are depicted in Table 1.

**Table 1:** Sociodemographic characteristics.

**Characteristic**	***n***	**%**
Gender		
Women	206	58.7
Men	145	41.3
Marital status		
Single	108	30.6
Married/relationship	227	65.0
Divorced/separated/widowed	16	4.6
Education		
≤ 12 years	155	44.2
12–15	117	33.3
>15	79	22.5
Religion		
Jewish	346	98.3
Muslim	2	0.6
Christian	2	0.6
Druze	1	0.3
Taking meds		
Yes	22	6.3
Metabolic health diagnoses		
None	340	96.9
1 or more	11	3.1
Mental health diagnoses		
None	291	82.9
1–2	56	14.0
3 or more	4	1.1
ISI		
No insomnia (<8)	103	29.3
Subclinical symptoms (8–13)	142	40.3
Moderate (14–21)	86	24.4
Severe (= >22)	21	6.0

### Instruments

#### Insomnia symptoms

The Insomnia severity index [ISI, (55)] is a 7-item questionnaire assessing the nature, severity, and impact of sleep difficulties in the last 2 weeks. A 5-point Likert scale is used to rate each item, with scores ranging 0–28 that yields four categories: absence of insomnia (0–7); subthreshold insomnia (8–14); moderate insomnia (15–21); and severe insomnia (22–28). The ISI has demonstrated 82.4% sensitivity, 82.1% specificity, and 82.2% agreement for detecting clinical insomnia with a cutoff score of 14 (56) and is sensitive to treatment outcomes (55). In this study the internal consistency was high (Macdonald's ω = 0.907).

#### Additional self-report sleep measures

Respondents were asked to report their typical bedtime, wake-up time, total sleep time (TST), and latency to fall asleep (SOL) in the past week.

#### Negative ruminations

The Ruminative Response Scale [RRS, (57, 58)] was used to assess ruminations with negative valence. The RRS is a self-report instrument describing intrusive negative cognitions in response to a depressed mood. The original instrument consisted of 22 items rated on a 4-point Likert scale ranging from 1 (never) to 4 (always). Subsequent psychometric analysis (58) distinguished between three subscales: Twelve depressed mood items (e.g., “I think about how alone I feel”; “I think about how hard it is to concentrate”), five brooding items (e.g., “I Think, why do I have problems other people don't have?”), and five reflection items with neutral valence (e.g., I Analyze recent events to try to understand why I am depressed”). The first two subscales are considered measures of negative rumination. As the RRS has not been previously validated in Hebrew, exploratory and confirmatory factor analyses (EFA and CFA, respectively) were performed to determine the factor structure. A single-factor structure had better fit statistics (see Supplementary Tables 1, 2) and was used in subsequent analyses. Macdonald's reliability of this ‘Negative Rumination' scale was ω = 0.961.

#### Positive ruminations

Responses to the Positive Affect questionnaire [RPA, (22)] was used to assess ruminations with positive emotional content. The RPA is comprised of seventeen items rated on a 4-point scale ranging from 1 (“almost never”) to 4 (“almost always”). The measure consists of three subscales: Dampening (e.g., “Remind yourself these feelings won't last”); Self-focused positive rumination (four items, e.g., “Think ‘I am achieving everything”'); and Emotion focused positive rumination (e.g., “Think about how happy you feel”). The questionnaire was translated by the authors and EFA and CFA were performed to validate the psychometric structure of the translation. A two-factor model had superior fit indices to the original three-factor structure. Factor 1 included items of the Emotion Focus and Self Focus subscales and one item from the Dampening subscale (“I think people will think I'm bragging”), the second factor included six of the eight items on the Dampening subscale (excluding the item loaded on factor 1 and “I think my streak of luck is going to end soon,” see Supplementary Tables 3, 4). These factors were used for further analyses: “Positive Rumination” and “Dampening” with Macdonald's ω reliabilities of 0.959 and 0.893, respectively.

#### Emotion regulation strategies

The Emotion Regulation Questionnaire [ERQ, (59)] was used to assess reappraisal and suppression emotion regulation tendencies. The ERQ is a ten-item self-report measure of two distinct strategies of emotion regulation: Reappraisal and suppression. As above, the Hebrew translation of the questionnaire has not been subjected to psychometric analysis. EFA and CFA revealed a two-factor structure that did not entirely overlap with the original item categorization, with two items cross-loading and therefore omitted from the final scales (Supplementary Table 5). The two final subscales of the ERQ had Macdonald's ω reliabilities of 0.861 for Reappraisal and 0.908 for Suppression.

### Procedure

The study was approved by the internal review committee of the authors' institution (authorization no. PsyE02052019.001). Ads were posted on social media and student forums, inviting respondents to answer a survey regarding sleep and emotions. The survey was generated and administered using Qualtrics software (Copyright © [2016], Qualtrics, Provo, UT). The survey opened with a brief description of the study, to which respondents were required to assent in order to continue.

### Statistical analysis

Data cleanup, descriptive statistics and moderation analysis were done in SPSS v28.0 (IBM SPSS, 2016, Armonk, NY). Mahalanobis Distance analysis was used to detect invalid responses (60) to the ISI, using the additional sleep measures (time in bed, calculated as the time difference between bedtime and wake-up time; TST and SOL) as predictors. Applying a criterion of *p* < =0.001 one respondent was removed. Factor analyses of newly translated instruments were run in JASP v0.14.1 (JASP Team, 2020). Statistical power was calculated using Analytics Calculators (61). EFAs were run using maximum likelihood estimation, with promax rotation and based on the correlation matrix on randomly selected 50% of the sample, and confirmed with CFA on the other 50%. Fit indices included the Tucker-Lewis Index (TLI ≥ 0.95), Comparative Fit Index (CFI > = 0.95), Root Mean Square Error of Approximation (RMSEA ≤ 0.8), and Standard Root Means Square of Residual (SRMR ≤ 0.08) (62, 63).

## Results

Descriptive statistics and correlations are depicted in Table 2 and in Supplementary Table 6. Gender correlated weakly with several measures indicating that in general women had worse sleep, reported more negative ruminations and dampening ruminations, less positive ruminations, and less suppression. Age weakly correlated with ISI and TST, with older respondents having higher ISI scores and lower TST. Additionally, older respondents reported getting up significantly earlier. Age did not correlate with any of the rumination and emotion regulation measures. As the correlations with ISI were weak, and gender or age differences were not predicted, these factors were not considered in further analyses. The number of physical health diagnoses weakly correlated with the ISI (*R* = 0.137, *p* = 0.010, Supplementary Table 6), and the number of mental health diagnoses negatively correlated with subjective sleep onset latency (SOL, *R* = −0.117, *p* = 0.028, Supplementary Table 6). Again, these correlations were weak, and beyond the scope of our predictions. Moreover, the number of participants with any physical health diagnoses was 11. Thus, these variables were not included in further analyses.

**Table 2:** Descriptive statistics and first-order correlations.

		***M (SD)***	**[1]**	**[2]**	**[3]**	**[4]**	**[5]**	**[6]**	**[7]**	**[8]**	**[9]**	**[10]**	**[11]**
[1]	Age	30.42 [6.01]	1										
[2]	Gender	0 = F |1=M	0.078	1									
[3]	ISI	11.39 [6.6]	0.113*	−0.145**	1								
[4]	Bedtime	23:37 [1:15]	−0.091	−0.037	0.043	1							
[5]	Wake time	7:17 [1:22]	−0.194**	0.060	−0.262**	0.558**	1						
[6]	TST	6.4 [1.33]	−0.121*	0.146**	−0.708**	−0.115*	0.472**	1					
[7]	SOL	35.51 [26.03]	−0.006	−0.121*	0.263**	0.121*	−0.012	−0.259**	1				
[8]	Reapp.	4.51 [1.15]	−0.045	0.041	−0.228**	0.052	−0.010	0.122*	−0.030	1			
[9]	Supp.	3.74 [1.75]	0.043	−0.179**	0.363**	−0.070	−0.151**	−0.359**	0.084	−0.397**	1		
[10]	NR	1.89 [0.71]	−0.092	−0.260**	0.567**	0.083	−0.100	−0.488**	0.151**	−0.392**	0.572**	1	
[11]	PR	1.99 [0.88]	−0.007	0.209**	−0.128*	−0.004	−0.014	0.133*	0.026	0.373**	−0.637**	−0.485**	1
[12]	Damp.	1.74 [0.7]	−0.026	−0.222**	0.519**	0.042	−0.074	−0.331**	0.021	−0.368**	0.425**	0.738**	−0.325**

ISI, insomnia severity index; TST, totals sleep time; SOL, sleep onset latency; Reapp., Reappraisal factor from EFA of the ERQ; Suppr., Suppression factor from EFA of the ERQ; NR, negative rumination factor from EFA of the Ruminative Response Scale; PR, positive rumination derived from EFA of the Response to Positive Affect; Damp., Dampening derived from the EFA of Response to Positive Affect; *p <0.05; **p <0.01.

Reappraisal negatively correlated with the Negative Rumination scale and with the Dampening scale, and positively correlated with the Positive Rumination scale. Reappraisal also had weak correlations with TST and ISI. Suppression had moderate positive correlations with the Negative Rumination scale and the Dampening scale, and a negative correlation with the Positive Rumination scale. Suppression also correlated with the TST and ISI. The Negative Rumination scale negatively correlated with TST and positively correlated with ISI. The Positive Rumination scale had weak positive correlations with TST and negative with ISI. The Dampening scale positively correlated with ISI and negatively with TST (Table 2).

**Figure 2:**
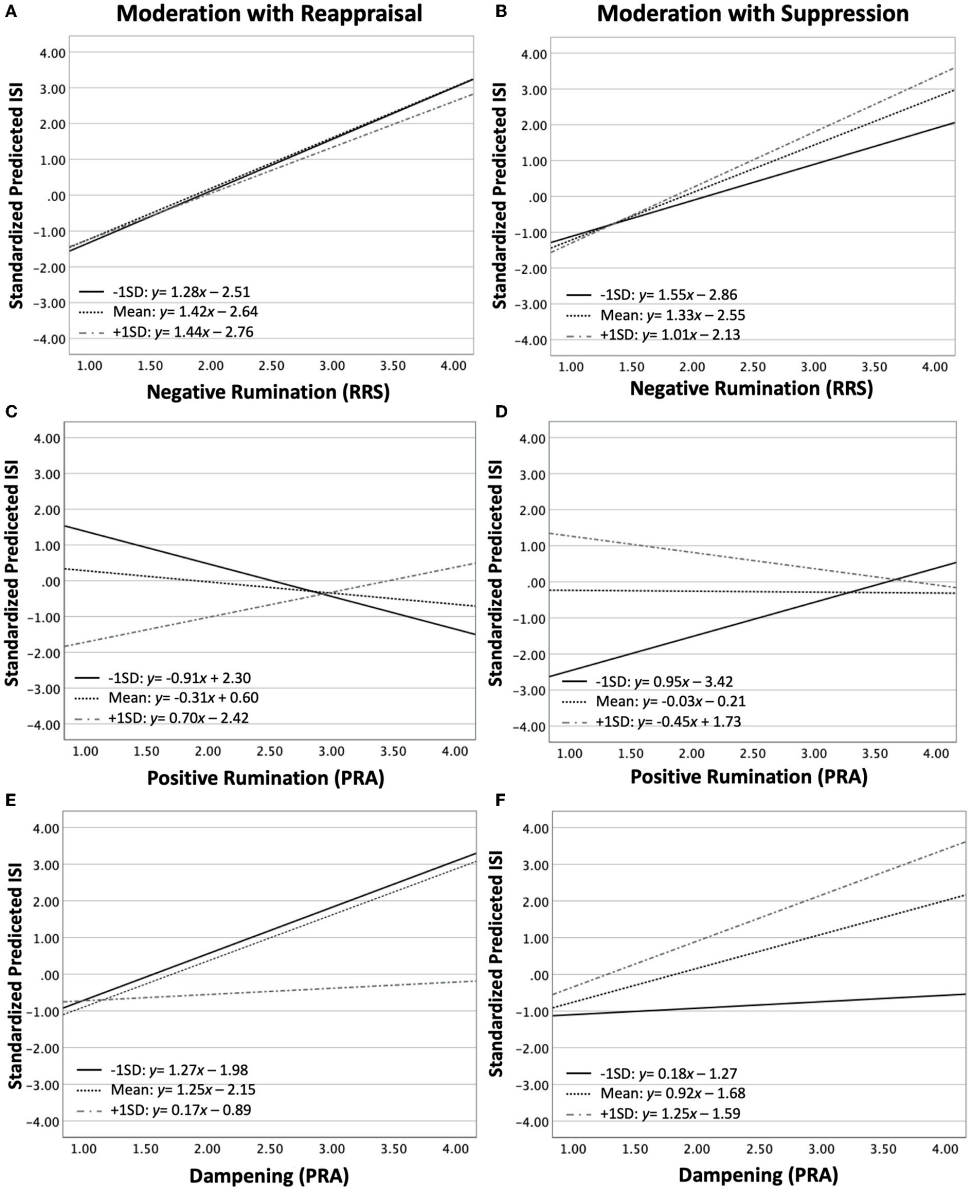
Testing the moderating effects of Reappraisal **(A,C,E)** and Suppression **(B,D,F)** on the association between Negative Rumination **(A,B)**, Postive Rumination **(C,D)** and Dampening **(E,F)** with insomnia Symptoms as reported on the ISI. The ordinate in each graph is the standardized predicted ISI derived from the moderation model for each rumination type. The lines depict the slope and intercept of the association between factors at three levels of the moderator: −1 standard deviation (SD, full black line), mean (hashed black line), +1 SD (hashed gray line).

Three hierarchical regression models were used to test moderation hypotheses, one model for each rumination type. In each model, rumination type (negative, positive or dampening) was entered in the 1^st^ step, both emotion regulation types (reappraisal and suppression) were entered in the 2^nd^, and interaction terms were entered in the 3^rd^. The moderation model for negative rumination is summarized in Table 3. As can be seen, the model was significant at all three steps, explaining about 31% of the variance across steps. In all three steps, negative rumination positively predicted ISI scores (step 1: *t* = 12.56, *p* < 0.001; step 1: *t* = 9.4, *p* < 0.001; step 1: *t* = 8.98, *p* < 0.001). The addition of Reappraisal and Suppression (step 2) and their interaction terms (step 3) was not significant, with nearly no change in the strength of the association between the Negative Rumination scale and ISI (Figures 2A,B).

**Table 3:** Moderation model estimates for the association between negative rumination and insomnia symptoms.

	**Model 1**				**Model 2**				**Model 3**			
	**B [SE]**	**β**	**CI 95%**	**VIF**	**B [SE]**	**β**	**CI 95%**	**VIF**	**B [SE]**	**β**	**CI 95%**	**VIF**
NR	3.685 [0.29]	0.56***	3.11, 4.26	1.00	3.46 [0.37]	0.52***	2.74, 4.19	1.56	3.35 [0.37]	0.51***	2.62, 4.09	1.62
Reap					0.04 [0.33]	0.01	−0.61, 0.69	1.24	0.10 [0.33]	0.02	−0.55, 0.76	1.28
Supp					0.41 [0.37]	0.06	−0.32, 1.13	1.60	0.49 [0.37]	0.07	−0.24, 1.21	1.64
NR * Reap									0.20 [0.36]	0.03	−0.51, 0.91	1.23
NR * Supp									0.63 [0.37]	0.08	−0.10, 1.37	1.25
Adjusted *R*^2^	0.309				0.308				0.309			
*F* Model	157.62***				52.84***				32.36***			
*R* change					0.002				0.006			
*F* change					0.63				1.44			
Cohen *F^2^*					N/A				N/A			

NR, negative rumination from RRS; Reap, Reappraisal from ERQ; Supp, Suppression from ERQ; Cohen F^2^ Effect size attributable to the addition of B, ***p < 0.001.

The moderation model for positive rumination is summarized in Table 4. The model was significant at all three steps, explaining 1.2% of the variance at step 1 (positive rumination alone), 15.4% in step 2, and 18.9% in step 3. In step 1, The Positive Rumination scale was negatively associated with ISI (step 1: *t* = −2.31, *p* = 0.021). The addition of Reappraisal and Suppression reversed this association (step 2: *t* = 3.16, *p* = 0.002). In step 2, Reappraisal had a small negative association with ISI (*t* = −2.04, *p* = 0.042) and suppression had a strong positive (*t* = 6.86, *p* < 0.001). The addition of interaction terms (step 3) eliminated the association between the Positive Rumination scale and ISI. Both interaction terms were significant in opposite directions (Positive Rumination ^*^ reappraisal: *t* = 2.34, *p* = 0.020; Positive Rumination ^*^ suppression: *t* = −2.27, *p* = 0.024). Thus, for lower values of reappraisal, positive rumination was negatively associated with ISI; whereas for higher levels of reappraisal, positive rumination was positively associated with ISI (Figure 2C). The opposite was observed for suppression: For lower levels of suppression, positive rumination was positively associated with ISI; whereas for higher levels of suppression, positive ruminations was negatively associated with ISI (Figure 2D).

**Table 4:** Moderation model for the association between positive rumination and insomnia symptoms.

	**Model 1**				**Model 2**				**Model 3**			
	**B [SE]**	**β**	**CI 95%**	**VIF**	**B [SE]**	**β**	**CI 95%**	**VIF**	**B [SE]**	**β**	**CI 95%**	**VIF**
PR	−0.92 [0.40]	−0.12*	−1.70, −0.14	1.00	1.52 [0.48]	0.20***	0.57, 2.47	1.71	0.86 [0.51]	0.12	−0.13, 1.63	2.01
Reap					−0.74 [0.36]	−0.11*	−1.45, −0.03	1.23	−0.87 [0.36]	−0.13	−1.59, −0.16	1.29
Supp					2.92 [0.43]	0.45***	2.08, 3.77	1.79	2.48 [0.44]	0.38***	1.62, 3.34	1.92
PR * Reap									0.86 [0.37]	0.13*	0.14, 1.59	1.25
PR * Supp									−0.96 [0.42]	−0.13*	−1.78, −0.13	1.35
Adjusted *R*^2^	0.012				0.154				0.189			
*F* Model	5.36*				22.27***				17.30***			
*R^2^* change					0.146				0.039			
*F* change					30.28***				8.41			
Cohen *F^2^*					0.17				0.05			

PR, positive rumination from PRA; Reap, Reappraisal from ERQ; Supp, Suppression from ERQ; Cohen F^2^ Effect size attributable to the addition of B, ***p < 0.001, *p < 0.050.

The moderation model for dampening is summarized in Table 5. The model was significant at all three steps, explaining 11.1% of the variance at step 1 (Dampening scale alone), 17.6% in step 2, and 19.0% in step 3. The Dampening scale positively predicted ISI scores in all three steps (step 1: *t* = 6.70, *p* < 0.001; step 1: *t* = 4.38, *p* < 0.001; step 1: *t* = 4.44, *p* < 0.001). In step 2, Suppression (*t* = 4.91, *p* < 0.001) was significant, but not reappraisal (*t* = −0.53, *p* = 0.598). The interaction term for Reappraisal was nearly significant (*t* = −1.89, *p* = 0.060), but not the interaction with suppression (*t* = 1.66, *p* = 0.098). Thus, higher levels of reappraisal attenuated the positive association between the Dampening scale and ISI (Figure 2E). The opposite was observed for suppression, although the effects were not significant (Figure 2F).

**Table 5:** Moderation model for the association between dampening and insomnia symptoms.

	**Model 1**				**Model 2**				**Model 3**			
	**B [SE]**	**β**	**CI 95%**	**VIF**	**B [SE]**	**β**	**CI 95%**	**VIF**	**B [SE]**	**β**	**CI 95%**	**VIF**
Damp	2.24 [0.33]	0.34***	1.57, 2.88	1.00	1.54 [0.35]	0.23***	0.85, 2.23	1.21	1.55 [0.35]	0.24***	0.86, 2.23	1.21
Reap					−1.91 [0.36]	−0.03	−0.90, 0.52	1.27	−0.13 [0.36]	−0.02	−0.85, 0.58	1.29
Supp					1.76 [0.36]	0.27***	1.05, 2.46	1.28	1.83 [0.36]	0.28***	1.13, 2.53	1.29
Damp * Reap									−0.61 [0.33]	−0.09	1.25, 0.03	1.07
Damp * Supp									0.59 [0.36]	0.08	−0.11, 1.29	1.06
Adjusted *R*^2^	0.111				0.176				0.190			
*F* Model	44.85***				25.84***				17.38***			
*R^2^* change					0.069				0.019			
*F* change					14.59***				4.01*			
Cohen *F^2^*					0.08				0.02			

Damp, Dampening from PRA; Reap, Reappraisal from ERQ; Supp, Suppression from ERQ; Cohen F^2^ Effect size attributable to the addition of B, ***p < 0.001, *p < 0.050.

Finally, we used Cumming's rule of thumb (64) to determine, for each model whether the strength of the association between the rumination type and ISI was significantly affected by the addition of Reappraisal, Suppression and interaction terms: Only for positive rumination the addition of reappraisal and suppression to the model significantly changed the association between the rumination type and insomnia symptoms (see Supplementary Figure 1).

## Discussion

In this study, we attempted to assess the possible effects of positive ruminations on subjective sleep quality, and the moderating role of reappraisal and suppression tendencies on the association between rumination and insomnia symptoms. While there is ample evidence indicating that negative ruminations interfere with sleep in both clinical and non-clinical populations [e.g., (10)], to our knowledge, the effects of positive ruminations on sleep complaints have not been previously examined in healthy participants. The rationale behind the tested models was that ruminations reflect reduced executive control on affect-inducing cognitions [e.g., (21)], that may lead to elevated arousal which interferes with sleep; whereas cognitive reappraisal and/or suppression constitute an attempt to exert executive control over these runaway repetitive thoughts, thereby potentially moderating the association between rumination and insomnia symptoms.

Insomnia symptoms were measured using the Insomnia Severity Index [ISI, (55)]. As this was a convenience sample and data were collected via online surveys, it is worth highlighting the associations between the ISI and self-reported quantitative measures of sleep. The strongest correlate of ISI scores was with sleep amount. In general the average amount of sleep was below the recommended 7–8 h, and respondents who reported sleeping less had higher scores on the ISI. The ISI also correlated with wakeup time but not with bedtime, suggesting that insufficient sleep opportunity was the main driver of insomnia symptoms rather than circadian misalignment. ISI scores also positively correlated with the time it took respondents to fall asleep, albeit weakly, suggesting that other factors, in addition to reduced sleep opportunity, may have contributed to respondents subjective sleep quality.

Consistent with our predictions, negative rumination and ruminatory dampening of positive affect were associated with more insomnia symptoms, whereas positive ruminations negatively correlated with insomnia symptoms. Contrary to our hypotheses however, negative rumination did not interact with either reappraisal nor suppression, indicating a lack of moderation. In fact, with negative rumination in the model, the associations between reappraisal or suppression with ISI scores were nullified. We found only one study that tested the moderating effects of reappraisal and suppression on negative rumination in an experimental setting. Witvliet et al., (65) asked participants to engage in either compassionate reappraisal or emotional suppression after ruminating about an offending event. They found that the two strategies were equivalent in reducing negative emotion measures and physiological arousal. Therefore, it remains possible that when used with intention, both reappraisal and suppression can attenuate negative affect induced by negative ruminations.

Potentially, our results reflect the statistical fact that the correlations of reappraisal and suppression scales with the negative rumination measure were larger than their correlations with ISI scores. Alternatively, the results suggest a shared mechanism as demonstrated by Aldao and Nolen-Hoeksema (66). In their study, a non-clinical sample of undergraduate students completed a survey regarding their symptoms of depression, anxiety and eating disorders, and their tendencies for negative rumination, reappraisal and suppression. Using structural equation modeling the authors showed that negative rumination, suppression and reappraisal all loaded on a single latent factor of cognitive emotion regulation, which in turn was related to all three types of psychopathology symptoms. Importantly, the authors found that negative rumination had a larger loading coefficient than the other components, suggesting that negative ruminations are more potent emotion regulators than suppression and reappraisal, when measured as behavioral tendencies that predict psychopathology symptoms.

Unlike negative rumination, positive rumination interacted with reappraisal and suppression. There was a weak positive association between positive rumination and ISI scores, consistent with findings the positive emotions improve sleep in healthy participants [e.g., (41)]. This association was inverted when reappraisal, suppression and their interaction terms were entered into the model. Reappraisal and suppression had opposite moderating effects such that among respondent with higher reappraisal scores, positive rumination was associated with *more* insomnia symptoms. In contrast, among respondent with higher suppression scores positive rumination was associated with *less* insomnia symptoms. In other words, reappraising positive events and emotions may increase sleep disturbance, whereas suppressing the bodily effects of these positive emotions may have calming effects and could facilitate sleep.

The model with dampening partially supported our hypotheses. Dampening was positively associated with insomnia symptoms similar to negative rumination and in line with evidence the dampening increases arousal (33). Reappraisal weakly (close to significant) moderated the association between dampening and ISI scores such that respondents with higher scores on the reappraisal scale had a diminished association between the two latter measures. Suppression was significant in the model but did not interact with dampening, although the direction of the effect was opposite to reappraisal, with lower suppression diminishing the association between dampening and ISI scores, and higher suppression enhancing the association. Nevertheless, the strength of association between dampening and ISI was not significantly changed by the addition of reappraisal and suppression to the model. Thus, dampening behaved similar to negative rumination, consistent with finding that dampening is associated with negative affect and depression symptoms (22, 28), which are themselves associated with sleep disturbances in non-clinical samples (67).

Few studies have assessed the role of trait reappraisal and suppression in sleep with inconsistent results: In a recent study in a non-clinical sample, Sella and Borella (68) found that more self-reported sleep difficulties were associated with more bedtime suppression and reappraisal thought control strategies. Some authors have found positive associations only between suppression and self-reported sleep difficulty and no association with reappraisal (69, 70); others found a negative association between reappraisal and sleep difficulty (71), and yet others report that neither factor was associated with sleep difficulty (72). Here we report that both strategies correlated, albeit weakly, with insomnia symptoms in the expected directions, consistent with observations from experimental [e.g., (73)] and observational studies [e.g., (74)]. With respect to their role as moderators, reappraisal and suppression mainly moderated the association between positive rumination and insomnia symptoms, with weak effects on dampening. The counter-intuitive effect, whereby among respondents with higher reappraisal positive rumination was associated with *more* insomnia symptoms, potentially indicates that reappraisal is a less efficient strategy to down regulate arousal associated with positive emotions, at least in the context of sleep.

These findings should be interpreted with caution. First, while the sample size was adequate for the statistical analyses, it is a single study (to our knowledge) on a relatively young sample, that addressed the associations between different rumination types, reappraisal and suppression strategies with measures of sleep disturbance. Future studies, with samples derived from other populations, including clinical samples, using additional methods, are required to generalize these findings. Moreover, the sampling method and the format of an online survey limited our ability to control for factors such as the profession of the respondents, their psychiatric diagnosis and use of medications. Second, in our analyses we did not control for age and gender, as this was not hypothesized and the associations with ISI were weak. However, both gender and age are factors in the prevalence of insomnia symptoms [e.g., (75)], and different mechanisms may be involved in sleep disturbances among different groups. Future studies, with larger samples, may allow for more detailed stratification. Third, several of the measures have not been independently validated in Hebrew, and while we applied psychometric analyses to these measures, their validity should be assessed independently. Fourth, we proposed a causal model in which insomnia symptoms were the dependent variable, however ample evidence exists that sleep difficulty predicts dysregulation of emotions and impaired executive functioning associated with reappraisal and suppression [e.g., (76)]. Therefore, there may be a reciprocal relationship that needs further investigation.

The abovementioned limitations notwithstanding, these findings may be of value both clinically and theoretically, as we believe we demonstrated that different types of emotion regulation strategies interact differently to impact sleep quality. The postulated function of emotion regulation strategies is to decrease negative and enhance positive emotions (77, 78). However, in the context of sleep enhancing positive emotions may result in elevated arousal that interferes with sleep. Specifically, reappraisal of positive emotions may prolong emotional arousal thereby interfering with subjective sleep quality, whereas suppressing positive emotions may reduce such arousing effects. We further demonstrated that the association negative rumination with sleep disturbance is robust, and not easily moderated by other emotion regulation strategies, which has implications for clinical intervention protocols. These insights may further inform future research on the antecedents and underlying mechanisms of sleep disturbances.

## Data availability statement

The datasets presented in this study can be found in online repositories. The names of the repository/repositories and accession number(s) can be found below: https://osf.io/zdu6x/?direct%26mode=render%26action=download%26mode=render.

## Ethics statement

The studies involving human participants were reviewed and approved by IH, Psychology Department, Tel Hai College, Meirav Hen, Psychology Department, Tel Hai College. The Ethics Committee waived the requirement of written informed consent for participation.

## Author contributions

IH was in charge of study design, analytic processing, and writing of the manuscript. LP and TC created the survey, collected data, conducted a preliminary data cleanup, and analysis. All authors contributed to the article and approved the submitted version.

## Conflict of interest

The authors declare that the research was conducted in the absence of any commercial or financial relationships that could be construed as a potential conflict of interest.

The reviewer MK declared a shared affiliation with the author IH to the handling editor at the time of review.

## Publisher's note

All claims expressed in this article are solely those of the authors and do not necessarily represent those of their affiliated organizations, or those of the publisher, the editors and the reviewers. Any product that may be evaluated in this article, or claim that may be made by its manufacturer, is not guaranteed or endorsed by the publisher.
