# Supplementary material for: Positive rumination can (also) interfere with sleep: A study in a non-clinical sample
Source: Front Psychiatry. 2022 Aug 9;13:889810. doi: 10.3389/fpsyt.2022.889810 (PMC9396259; doi:10.3389/fpsyt.2022.889810)
Supplement: Supplementary file 1 [file Data_Sheet_1.docx]

**Supplemental Materials**

[EFA and CFA on the Rumination and Reflection Scale 2](#_Toc104909770)

[EFA and CFA on the Responses to Positive Affect Scale 4](#_Toc104909771)

[EFA and CFA on the Emotion Regulation Questionnaire 7](#_Toc104909772)

[Table 6: Full Correlation Table 9](#_Toc104909773)

[Figure 1: Cumming’s Rule of Thumb Comparison of Rumination Coefficients. 10](#_Toc104909774)

EFA and CFA on the Rumination and Reflection Scale (Nolen-Hoeksema & Morrow, 1991; Treynor et al., 2003)

An EFA was run on a random selection of 50% of respondents using maximum likelihood estimation, with promax rotation and based on the correlation matrix. The analysis yielded a single factor which included nine of the ten items (item “I write down what you are thinking about and analyze it” was excluded). The factor explained 50.1% of the variance, with KMO=.907, with significant Bartlett’s sphericity test p<.001. To determine which of the two models - the original two-factor or single factor from the EFA - performed better, CFA was run with each model, on the other half of the sample. The original model was mediocre, but the single-factor structure had acceptable fit indices (Table 1), and thus the this structure was used for further analyses. Based on this analysis, a composite Positive Rumination (PR) scale was used for subsequent analyses.

| Table 1: Comparison of goodness of fit measures for two CFA models of the RRS | | | | | | |
| --- | --- | --- | --- | --- | --- | --- |
| Model | 𝛘^2^ | *df* | CFI | TLI | RMSEA [CI90%] | SRMR |
| Original 2 factor | 108.59** | 34 | 0.92 | 0.89 | 0.113 **  [0.09, 0.14] | 0.063 |
| Single factor from EFA | 78.56** | 27 | 0.94 | 0.92 | 0.105 **  [0.08, 0.13] | 0.048 |
| *Note:* CFI, comparative fit index; TLI, Tucker-Lewis index; RMSEA, root mean square error of approximation; SRMR, standardized root mean square residual; ** = *p*<.001 | | | | | | |

| Table 2: Confirmatory factor analysis of the Rumination and Refection Scale (RRS) | | |
| --- | --- | --- |
|  | B (SE) | β |
| (B) think “What am I doing to deserve this?” | 0.852 (0.065) | 0.827 |
| (R) analyze recent events to try to understand why you are depressed | 0.75 (0.065) | 0.764 |
| (B) think “Why do I always react this way?” | 0.759 (0.063) | 0.786 |
| (R) go away by yourself and think about why you feel this way | 0.367 (0.052) | 0.518 |
| (B) think about a recent situation, wishing it had gone better | 0.675 (0.061) | 0.743 |
| (B) think “Why do I have problems other people don’t have?” | 0.812 (0.064) | 0.816 |
| (B) think “Why can’t I handle things better?” | 0.746 (0.062) | 0.784 |
| (R) analyze your personality to try to understand why you are depressed | 0.63 (0.058) | 0.732 |
| (R) go someplace alone to think about your feelings | 0.368 (0.053) | 0.511 |
| (B) think “What am I doing to deserve this?” | 0.852 (0.065) | 0.827 |
| **Macdonald’s ω** | 0.961 |  |
| *Note*: Unstandardized with standard error in brackets, and standardized coefficients for each item. (B) = brooding subscale; (R) = reflection subscale | | |

EFA and CFA on the Responses to Positive Affect Scale (Feldman et al., 2008)

An EFA was run on a random selection of 50% of respondents using maximum likelihood estimation, with promax rotation and based on the correlation matrix. The model yielded two factors that explained 62.3% of the variance, with KMO=.908, with significant Bartlett’s sphericity test p<.001. Factor 1 included all the items of the Emotion Focus and Self Focus subscales, and one item from the Dampening subscale (“I think people will think I’m bragging”), the second factor included six of the eight items on the Dampening subscale (excluding the item loaded on factor 1 and “I think my streak of luck is going to end soon”). Based on these results, two CFA models were tested, on the second half of the sample, one of the original factor cluster the second of the two-factor structure from the EFA. Both models were mediocre, however fit variables were slightly better for the two-factor structure derived from the EFA (Table 3), and thus the this structure was used for further analyses (Table 4). Based on this analysis, two subscales of the RPA were derived for Positive Rumination (PR) and Dampening with Macdonald’s ω reliabilities of 0.959 and 0.893, respectively.

| Table 3: Comparison of goodness of fit measures for two CFA models of the RPA | | | | | | |
| --- | --- | --- | --- | --- | --- | --- |
| Model | 𝛘^2^ | *df* | CFI | TLI | RMSEA  [CI 90%] | SRMR |
| Original three-factor | 333.27** | 116 | 0.91 | 0.89 | 0.104  [0.09, 0.12] | 0.152 |
| EFA Two-factor | 291.34** | 103 | 0.92 | 0.91 | 0.103  [0.09, 0.12] | 0.081 |
| *Note:* CFI, comparative fit index; TLI, Tucker-Lewis Index; RMSEA, root mean square error of approximation; SRMR, standardized root mean square residual; ** = *p*<.001 | | | | | | |

| Table 4: Confirmatory factor analysis of the Responses to Positive Affect (RPA) Scale | | | | |
| --- | --- | --- | --- | --- |
|  | Factor 1  Positive Rumination | | Factor 2 - Dampening | |
|  | B | β | B | β |
| I think how happy I feel | 0.88 (0.060) | 0.883 |  |  |
| I think how strong I feel | 0.954 (0.062) | 0.905 |  |  |
| I think about how I feel up to doing everything | 1.005 (0.064) | 0.913 |  |  |
| I notice how I feel full of energy | 0.923 (0.062) | 0.885 |  |  |
| I savour the moment | 0.779 (0.067) | 0.755 |  |  |
| I Think “people will think I’m bragging” | 0.471 (0.057) | 0.584 |  |  |
| I Think “I am achieving everything” | 0.900 (0.066) | 0.841 |  |  |
| I Think “I am living up to my potential” | 0.89 (0.068) | 0.821 |  |  |
| I Think about how proud I am of myself | 0.984 (0.069) | 0.867 |  |  |
| I Think “I am getting everything done” | 0.979 (0.067) | 0.88 |  |  |
| **Macdonald’s ω** | 0.96 |  |  |  |
| I Think “I don’t deserve this” |  |  | 0.473 (0.056) | 0.609 |
| I Think about things that could go wrong |  |  | 0.734 (0.056) | 0.838 |
| I Think about things that have not gone well for me |  |  | 0.79 (0.057) | 0.873 |
| I Remind myself these feelings won’t last |  |  | 0.559 (0.057) | 0.68 |
| I Think “This is too good to be true” |  |  | 0.525 (0.056) | 0.662 |
| I Think about how hard it is to concentrate |  |  | 0.673 (0.062) | 0.735 |
| **Macdonald’s ω** |  |  | 0.89 |  |
| Unstandardized with standard error in brackets, and standardized coefficients for each item. The covariance between the suppression was *R*=-0.42. | | | | |

EFA and CFA on the Emotion Regulation Questionnaire (Gross & John, 2003)

An EFA was run on a random selection of 50% of respondents using maximum likelihood estimation, with promax rotation and based on the correlation matrix. The model yielded two factors that explained 61.4% of the variance, with KMO=.814, with significant Bartlett’s sphericity test p<.001. However, the factor structure did not entirely overlap with the original item categorization, with two reappraisal items (“When I want to feel more positive emotion (such as joy or amusement), I change what I’m thinking about” and “When I want to feel less negative emotion (such as sadness or anger), I change what I’m thinking about”) cross-loading with the suppression factor. Thus, these two items were eliminated in the CFA. The model yielded mediocre goodness of fit measures (Table 5), with covariance between the reappraisal and suppression *R*=-0.45. The chi-square value was significant (𝛘^2^_19_ = 45.51, *p* < .001), but this is to be expected in larger samples, where small discrepancies between the observed and predicted matrixes may become statistically significant. Based on this analysis, two subscales of the ERQ were derived for Reappraisal and Suppression with Macdonald’s ω reliabilities of 0.861 and 0.908, respectively.

| Table 5: Confirmatory factor analysis of the Emotion Regulation Questionnaires (ERQ) | | | | |
| --- | --- | --- | --- | --- |
|  | Factor 1 - Reappraisal | | Factor 2 - Suppression | |
|  | B | β | B | β |
| When I’m faced with a stressful situation, I make myself think about it in a way that helps me stay calm. | 1.022 (0.098) | 0.722 |  |  |
| When I want to feel more positive emotion, I change the way I’m thinking about the situation. | 1.008 (0.082) | 0.814 |  |  |
| control my emotions by changing the way I think about the situation I’m in. | 1.19 (0.088) | 0.866 |  |  |
| When I want to feel less negative emotion, I change the way I’m thinking about the situation. | 0.939 (0.088) | 0.731 |  |  |
| **Macdonald’s ω** | 0.861 |  |  |  |
| I keep my emotions to myself. |  |  | 1.228 (0.118) | 0.71 |
| When I am feeling positive emotions, I am careful not to express them. |  |  | 1.437 (0.12) | 0.786 |
| When I am feeling negative emotions, I make sure not to express them. |  |  | 1.796 (0.12) | 0.911 |
| I control my emotions by not expressing them. |  |  | 1.829 (0.133) | 0.861 |
| **Macdonald’s ω** |  |  | 0.908 |  |
| *Note:* Unstandardized with standard error in brackets, and standardized coefficients for each item. Goodness of fit measures: TLI = .91; CFI = 0.94; RMSEA = .120 [CI 90% .09-0.15]; SRMR = .07; 𝛘^2^ (19) = 66.37, *p* < .001. The covariance between the reappraisal and suppression was *R*=-0.47. | | | | |

## Table 6: Full Correlation Table


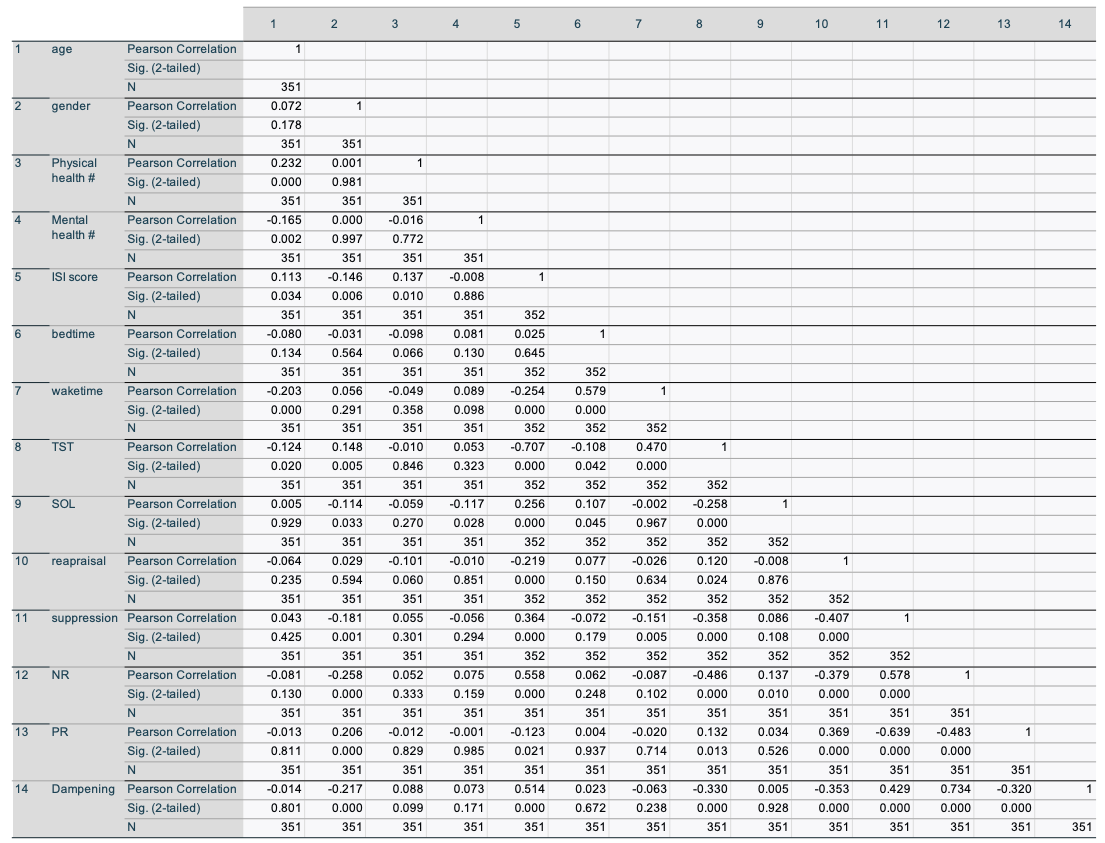


*Note*: Physical Health, # of endorsed physical health diagnoses; Mental Health, # of endorsed mental health diagnoses; ISI, insomnia severity index; TST, totals sleep time; SOL, sleep onset latency; Rapp., Reappraisal factor from EFA of the ERQ; Suppr., Suppression factor from EFA of the ERQ; NR, negative rumination factor from EFA of the Ruminative Response Scale; PR, positive rumination derived from EFA of the Response to Positive Affect; Damp., Dampening derived from the EFA of Response to Positive Affect.

## Figure 1: Cumming’s Rule of Thumb Comparison of Rumination Coefficients.


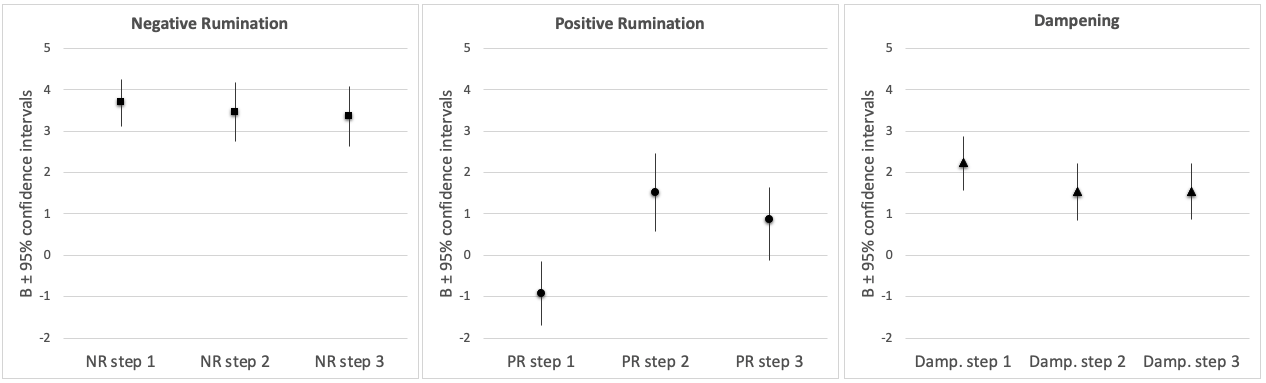


Figure 1: unstandardized coefficients and 95% confidence intervals for rumination type in each model. According the Cumming’s^[[1]](#footnote-1)^ rule of thumb, an overlap of 50% or less indicates statistically significant difference between estimates in the regression model. Thus, only for positive rumination the addition reappraisal and suppression to the model significantly change the association between the rumination type and insomnia symptoms (measured with ISI).

1. Cumming, Geoff. “Inference by Eye: Reading the Overlap of Independent Confidence Intervals.” *Statistics in Medicine* 28, no. 2 (2009): 205–20. [↑](#footnote-ref-1)
